# The Methodology of Adaptive Levels of Interval for Laser Speckle Imaging

**DOI:** 10.3390/jimaging10110289

**Published:** 2024-11-11

**Authors:** Ali A. Al-Temeemy

**Affiliations:** 1 Laser and Optoelectronics Engineering Department, College of Engineering, Al-Nahrain University, Baghdad 64040, Iraq; ali.a.al-temeemy@nahrainuniv.edu.iq or ali.al-temeemy@liverpool.ac.uk; 2 Electrical Engineering and Electronics Department, Faculty of Science and Engineering, University of Liverpool, Liverpool L69 3GJ, UK

**Keywords:** adaptive levels of interval, laser speckle imaging, dynamic speckle processing, laser imaging system

## Abstract

A methodology is proposed for use in the laser speckle imaging field. This methodology modified the graphical and numerical speckle pattern imaging methods to improve their extraction and discrimination capabilities when processing the embedded temporal activity in the images of laser speckle patterns. This is through enabling these methods to adapt the levels of speckle images’ interval during processing to speed up the process and overcome the lack of discrimination when they deal with a complex scattering medium having regions of various scales of activity. The impact of using the new methodology on the imaging methods’ performance was evaluated using graphical and numerical evaluation tests, in addition, an exceptional laser speckle imaging system was designed and implemented to undertake a series of experimental validation tests on this methodology. The evaluation and experimental validation tests show the effectiveness of this methodology on the extraction and discrimination capabilities for the standard imaging speckle pattern methods and prove its ability to provide high performance with the real images of speckle patterns. The results also show an improvement in the processing speed for both graphical and numerical methods when the adaptive levels methodology is applied to them, which reaches 78% for the graphical and 87% for the numerical speckle processing methods.

## 1. Introduction

Imaging the speckles of the laser light scattering and processing their patterns to extract spatial and temporal information with high resolution is known as laser speckle imaging [1,2]. It is gaining popularity in optical metrology and has emerged as a non-destructive, wide-field, and powerful optical technique in the laser imaging field [3,4,5]. Speckle images are random granular shape patterns with nonuniform illumination generated from illuminating diffuse surfaces with a laser source of high coherence degree [6,7,8,9]. These patterns previously considered noise need filtering, but the speckle imaging technique makes them a source of information [10].

The ability of laser speckle imaging to measure the activity information from the samples using indirect indexes makes it used by researchers in a wide range of application areas because it overcomes the time consumption, human intervention, and destructive tests rely on chemical and physical approaches [9,10,11,12,13]. Speckle imaging has been used in industrial applications to obtain topography and roughness for the objects’ surfaces, and also to monitor the temporal changes in the material shapes and their surfaces, such as absolute position, deformation, and other phenomena related to mass loss like drying, oxidation, and evaporation [14,15,16,17]. Regarding non-industrial applications, speckle imaging was used in the food industry for quality evaluation during collection, storage and transportation [13,18,19,20,21,22], in agriculture for plant seed, tissue, and growth assessment [13,23,24,25], in medicine to determine teeth erosion, characterize the dental material, burn surgery, dermatology, measurements of eye tremor, coagulation and blood flow [8,13,26,27,28,29,30,31].

Speckles can be categorized into objective speckles observed in free space or subjective speckles that result from scattered wavelets interference within the resolution of the imaging system [7,32]. These wavelets are reflected from the rough surface of height variations larger than the laser wavelength and have at each interference location different phases and magnitudes due to travelling along random paths to the image plane [6,7,32]. The speckle patterns consist of dark and bright areas, and the intensities for their images are determined at each pixel by the superposition of all the arrived wavelets at that pixel, where zero and maximum intensities occur if the scattered wavelets arrive at that pixel out-off phase and in phase respectively [7,33]. If the laser is stable and the scatters do not move within the medium, static speckle patterns result and do not change over time [2,33]. Dynamic speckle patterns occur when the scatters move within their medium, which makes the interference patterns change over time as a result of the Doppler shift of the interacting wavelets with these scatters and this makes the images for the dynamic speckle patterns carry spatiotemporal information [2,8,10,33,34].

The advancement in sensing technology for registering the successive intensity images for the interference patterns makes the intensity-based graphical and numerical approaches preferable for processing speckle images [5,9,11,17,35]. These approaches utilize the parameter of the time-variant intensity of the correlated dynamic speckle patterns to extract the temporal information from them [11,18,36]. The graphical approach is used to present the processing outcomes as a map of activity, while the numerical approach is usually adopted to quantify the activity or level of changes in specific speckle regions [35].

The methods of the graphical approach mainly determined the activity map by processing consecutive or non-consecutive speckle images, where the same speckle images are used multiple times during the process. An activity map is a 2D map of predefined feature values that is used to characterize the level of activity for the dynamic speckle patterns register as intensity images [11,35,37]. At each point in this map, the feature value can be obtained from the intensity values corresponding to the same pixel location in the registered images of the speckle patterns [11]. This map represents feature values of the ongoing process speed as gray levels of a specific number and can be displayed as a pseudo colour to characterize the activity areas efficiently within the scattering medium [11,18,36].

Consecutive methods like inertial moment, Fujii and many others process the speckle images at predefined time lag efficiently if the activity across the scattering medium has a specific time scale and time lag is adjusted accurately with it [11,12,18,35,38,39]. This requires pre-knowledge about the activity time scale in the monitored medium and an accurate time lag adjustment, which restricts the monitoring performance [33]. If the scattering medium has various time scales of activity in its regions, the non-consecutive methods can be used like temporal standard deviation and generalized difference, but this requires high computational processing power and the resultant activity map does not correspond to the activity order which affects the discrimination ability [14,17,35,40,41,42].

It is possible to obtain numerical outcomes from graphical methods rather than using methods of numerical approach when activity quantification is required. This procedure is not preferable because it is time-consuming and restricted to the number of gray levels used to represent the activity map, unlike the numerical methods that provide the numerical outcomes efficiently and with less computational power like the moments of the average value of differences method [35]. These methods determine the numerical outcomes by analysing the Temporal History of the Speckle Pattern (THSP) constructed from a few selected points of the speckle images [35]. Since the THSP construction is based on consecutive speckle images, similar restrictions for the graphical approach are associated with the numerical approach [12,35].

To handle the constraints described in both approaches, a novel methodology has been developed for processing images of speckle patterns, called the “Adaptive Levels of Interval (ALI) Methodology”. This methodology does not focus on developing the intensity analysis for the graphical and numerical approaches like the major research in literature but instead, it utilizes a robust and novel mechanism I have recently developed to improve approaches’ performance through deriving mathematical equations required to modify their processing algorithms [33]. This methodology enables both the graphical and numerical processing methods to adapt the levels of speckle images’ interval during processing to overcome the lake of discrimination with faster processing when they deal with a scattering medium having regions of various activity scales.

In the following sections, the adaptive levels of interval (ALI) methodology is introduced with an explanation about its mechanism. The graphical and numerical speckle processing methods are then presented, with a detailed explanation about the modification of these methods using the proposed methodology, as well as the graphical and numerical evaluation procedures and results. Experimental validation is then provided, including a description about the design and implementation of an exceptional laser speckle imaging system and an explanation of how this system can be used to undertake a series of experimental validation tests on ALI methodology. The results of experimental validation are then presented followed by discussion and conclusion to the presented work.

## 2. Adaptive Levels of Interval (ALI) Methodology

The presented methodology enables the graphical and numerical methods to process the information of temporal activity embedded in a speckle images sequence at eclectic levels of interval. It then combines graphical or numerical outcomes for these methods at all levels to improve their extraction and discrimination capabilities.

**Figure 1:**
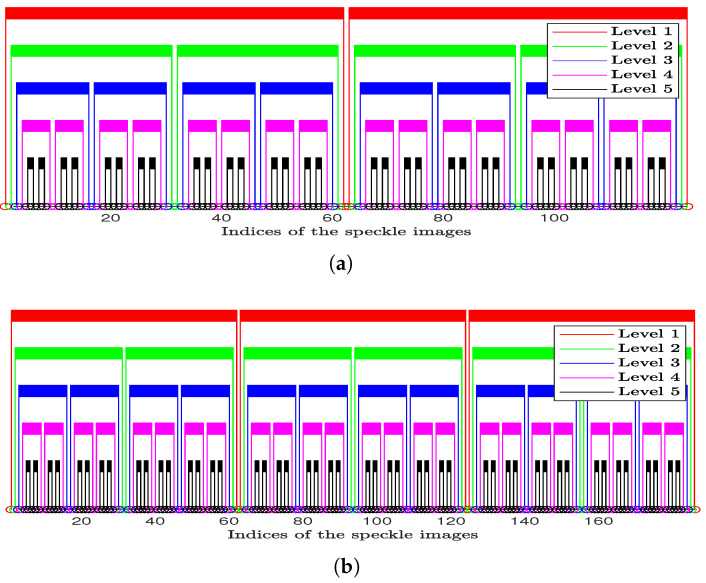

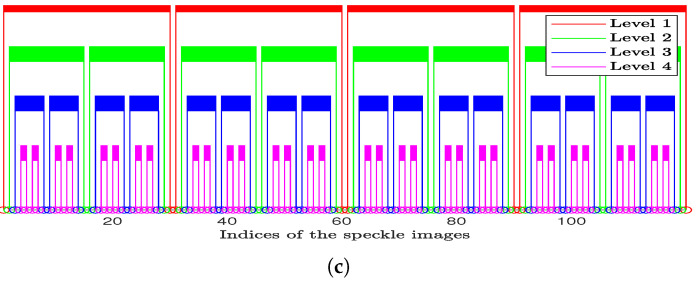
Principle of the indices’ selection mechanism for ALI methodology. (**a**) Five Levels of Interval with a sequence of 124 speckle images. (**b**) Five Levels of Interval with a sequence of 191 speckle images. (**c**) Four Levels of Interval with a sequence of 124 speckle images.

Referring to Figure 1 which shows a sequence of speckle image indices arranged as sets of defined image pairs (presented as connected indices in different colours). These sets of image pairs are defined by the levels of interval, where each one of them belongs to a specific level and its pairs have the same value of interval (presented in Figure 1 with a unique colour to discriminate them from the pairs of other levels).

The pairs’ indices determination in this methodology is based on a robust and novel mechanism. At each level, this mechanism selects the speckle image pairs in a way that makes the outcomes for the graphical or the numerical methods have different sensitivity scales with respect to the activity of the ongoing process and require less computational time. This is through adapting the pairs’ intervals (between their indices) and organising them using a non-overlapping structure between the pairs of different levels of interval to evade redundant usage of the speckle images with these methods.

Figure 1 presents two scenarios to demonstrate the principle of the indices’ selection mechanism for the proposed methodology. Figure 1a,b presents the first scenario of processing two different lengths of image sequences (124 and 191 speckle images) with the same levels of interval number (five levels). The second scenario of processing two sequences with the same length (124 speckle images) using different levels’ numbers (5 and 4 levels) is presented in Figure 1a and Figure 1c respectively.

The theoretical background of the graphical and numerical speckle processing methods is given in the following sub-sections, with a detailed explanation of the mathematical derivation used to modify the processing algorithms for these methods using the presented methodology.

### 2.1. Graphical Methods

Irrespective of the graphical methods used for processing speckle imaging, the proposed methodology starts by determining at each level *L* the activity map using the “time-variant intensity analysis approach” for the graphical method needs to be improved using the following general formula:

(1) $$A\left(x,y,L\right)=\frac{\sum_{K=1}^{\left\lfloor \frac{N}{T(1)}\right\rfloor 2^{L-1}+\left\lfloor \max\left(0,\frac{\left[NmodT\left(1\right)\right]+2-2^{L}}{T(L)}\right)\right\rfloor }\Delta \left(I_{a_{L,k}}\left(x,y\right),I_{b_{L,K}}\left(x,y\right)\right)}{\left\lfloor \frac{N}{T(1)}\right\rfloor 2^{L-1}+\left\lfloor \max\left(0,\frac{\left[NmodT\left(1\right)\right]+2-2^{L}}{T(L)}\right)\right\rfloor }$$

where L=1,2,⋯,M and the levels of interval number $M\leq \left\lfloor \log_{2}\left(1+N/2\right)\right\rfloor \in Z^{+}$, where this range can be used to adjust the responsivity. *N* is the speckle images number, and T(L) is the interval between images’ pairs at each level *L*, which can be determined using the following equation:

(2) $$T(L)=\{\begin{array}{cc} (2^{M+1}-2), & L=1\\ & \;L=1,2,\ldots ,M\\ (T(L-1)/2)-1, & L>1 \end{array}$$

Δ is the “time-variant intensity analysis approach” which is a function of the intensities I(x,y) values for speckle images of indices $\left[a_{L,K},b_{L,K}\right]$ at (x,y) coordinates in the monitored plane. These indices can be obtained using the following equation:

(3) $$[a_{L,K},b_{L,K}]=\{\begin{array}{cc} \left[L,L+T(L)-1\right] & K=1\\ \left[b_{L,K-1}+\tau (u_{L,K})+1,b_{L,K-1}+\tau (u_{L,K})+T(L)\right] & K>1 \end{array}$$

where,

(4) $$\begin{array}{ccc} u_{L,K} & = & \left[\left(1+sgn\left(-\left[\left(K-1\right)mod2^{L-1}\right]\right)\right)2^{L-1}\right]\\ & + & \left[\left(K-1\right)mod2^{L-1}\right] \end{array}$$

and the shift values are equal to:

(5) $$\tau \left(n\right)=2\sum_{K=2}^{M}\left(1-sgn(\left[n\;mod\left(2^{k-1}\right)\right])\right)\;,n=1,2,\ldots ,2^{M-1}$$

The methodology then combines the resultant activity maps to determine the final activity map AI(x,y) for all levels using the weighted equation:

(6) $$\begin{array}{cc} \upsilon {\left(x,y\right)}_{n}= & A\left(x,y,n+1\right)+\upsilon {\left(x,y\right)}_{n-1}\times \left(1-A(x,y,n+1)\right)\\ \; & \upsilon {\left(x,y\right)}_{o}=A\left(x,y,1\right),\;AI\left(x,y\right)=\upsilon {\left(x,y\right)}_{M-1} \end{array}$$

The weighted equation prioritises the high-sensitivity maps to the fast-fluctuation with greater weight values than the maps of slow-fluctuation sensitivity to improve the responsivity for the selected graphical method and reduce aliasing impact [33].

#### 2.1.1. Inertia Moment Method

This graphical method implements the consecutive calculation of the Inertial Moment. It extracts the activity map for each pixel by adding the square differences at that pixel’s location for a sequence of N images, where the original equation for Inertia Moment (I.M.) method can be described by [33,35,39].

(7) $$I.M.\left(x,y\right)=\frac{1}{N-1}\sum_{k=1}^{N-1}{\left(I_{k}\left(x,y\right)-I_{k+1}\left(x,y\right)\right)}^{2}$$

where $I_{k}\left(x,y\right)$ is the speckle image intensity value at frame index *k* and pixel location (x,y).

Referring to Equations (1) and (7), the activity map of the modified I.M. version at level of interval *L* derived from using the proposed methodology is defined as:

(8) $$A_{I.M.}\left(x,y,L\right)=\frac{\sum_{K=1}^{\left\lfloor \frac{N}{T(1)}\right\rfloor 2^{L-1}+\left\lfloor \max\left(0,\frac{\left[NmodT\left(1\right)\right]+2-2^{L}}{T(L)}\right)\right\rfloor }{\left[I_{a_{L,k}}\left(x,y\right)-I_{b_{L,K}}\left(x,y\right)\right]}^{2}}{\left\lfloor \frac{N}{T(1)}\right\rfloor 2^{L-1}+\left\lfloor \max\left(0,\frac{\left[NmodT\left(1\right)\right]+2-2^{L}}{T(L)}\right)\right\rfloor }$$

#### 2.1.2. Fujii Method

Like the I.M. method, the Fujii graphical method uses consecutive image processing for activity map generation. It determines the activity values for each pixel in this map using the total values of normalized absolute differences of intensity between all the consecutive images at that pixel’s location, where the original equation for (Fujii) method can be defined by [12,17,18,24,33,38]:

(9) $$Fujii\left(x,y\right)=\frac{1}{N-1}\sum_{k=1}^{N-1}\frac{|I_{k}\left(x,y\right)-I_{k+1}\left(x,y\right)|}{I_{k}\left(x,y\right)+I_{k+1}\left(x,y\right)}$$

Referring to Equations (1) and (9), the activity map of the modified Fujii version at level of interval *L* derived from using the proposed methodology is defined as:

(10) $$\begin{array}{cc} A_{Fujii}\left(x,y,L\right)=\frac{\sum_{K=1}^{\left\lfloor \frac{N}{T(1)}\right\rfloor 2^{L-1}+\left\lfloor \max\left(0,\frac{\left[NmodT\left(1\right)\right]+2-2^{L}}{T(L)}\right)\right\rfloor }\left[\frac{|I_{a_{L,k}}\left(x,y\right)-I_{b_{L,K}}\left(x,y\right)|}{I_{a_{L,k}}\left(x,y\right)+I_{b_{L,K}}\left(x,y\right)}\right]}{\left\lfloor \frac{N}{T(1)}\right\rfloor 2^{L-1}+\left\lfloor \max\left(0,\frac{\left[NmodT\left(1\right)\right]+2-2^{L}}{T(L)}\right)\right\rfloor } & \end{array}$$

**Figure 2:**
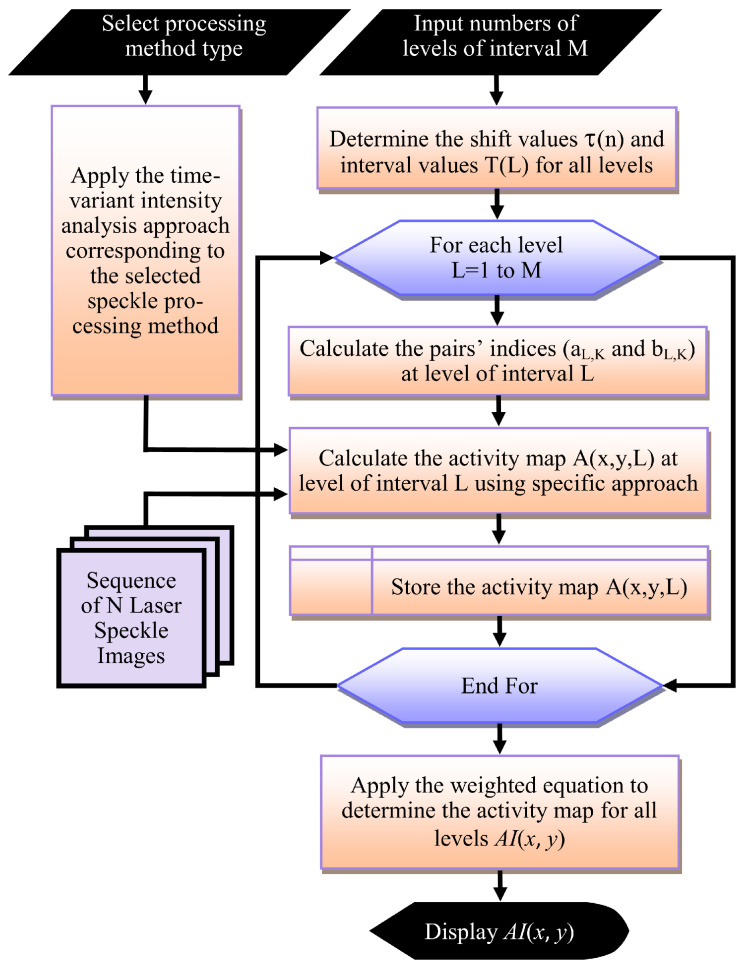
The general steps used by the proposed adaptive levels of interval methodology to improve the performance of the graphical methods.

Figure 2 summarises the general steps used by the adaptive levels of interval methodology to improve the graphical methods’ performance. These general steps comprise:

1. Select the type of graphical processing method I.M., Fujii, etc. (used to extract the activity from *N* speckle images) and identifying the level of interval number *M* used which should not exceed $\left\lfloor \log_{2}\left(1+N/2\right)\right\rfloor \in Z^{+}$.
2. Determine the shift values τ(n) and the interval value T(L) for all levels using Equations (5) and (2) respectively. These values are calculated once and then used repeatedly in the pairs’ indices ($a_{L,K}$ and $b_{L,K}$) calculation step.
3. Calculate the activity map A(x,y,L) for the selected graphical method at each level of interval *L* by performing the following operations:
  - Using the pre-calculated shift and interval values with Equations (3) and (4) to calculate the pairs’ indices ($a_{L,K}$ and $b_{L,K}$) at level of interval *L*.
  - Applying the “time-variant intensity analysis approach” corresponding to the selected graphical method on activity map Equation (1) to determine the activity map at *L* using the image pairs of the indices ($a_{L,K}$ and $b_{L,K}$) calculated in the previous step.
  - Storing the calculated map at a level of interval *L* temporarily for further calculations.
4. Apply the general weighted Equation (6) to find the activity map AI(x,y) for the selected graphical method. This map can then be stored and displayed.

### 2.2. Numerical Methods

Regarding the numerical methods, the presented methodology improves these methods by constructing the THSP at different levels of interval. It then analyses the resultant THSPs using the co-occurrence matrix with any numerical method’s algorithms like “moments of the average value of differences” (AVD). The resultant numerical outcomes for all levels are then combined using the weighted equation to quantify the activity.

THSP is a two-dimensional integer matrix representing the time history of the speckle pattern and has values between 0 and 255 [35]. This matrix is constructed from randomly selected points of the consecutive speckle image sequence, where the points’ locations follow a Gaussian distribution of pre-defined parameters (mean and standard deviation values) [35]. Instead of the consecutive approach which uses the whole speckle image sequence, the proposed methodology constructs the THSP_*L*_ at each level *L* from a set of image pairs of that sequence to make the resultant THSP matrices have different sensitivity scales with the ongoing process activity.

The co-occurrence Matrix (COM) is a two-dimensional intermediary matrix of size equal to 256 by 256, used to analyse the dispersion (transition histogram of intensity) of the THSP matrix of Q random points and N speckle image samples. This COM_*L*_ matrix can be calculated for each THSP_*L*_ of level *L* using the following equation [35]:

(11) $${COM}_{L}(i,j)=\sum_{q=1}^{Q}\sum_{k=1}^{N-1}=\{\begin{array}{cc} 1,\; & \mathrm{if}\;{\mathrm{THSP}}_{L}(q,k)=i\\ & \mathrm{and}\;{\mathrm{THSP}}_{L}(q,k+1)=j\\ 0, & \mathrm{otherwise} \end{array}$$

The resultant COM matrices can be then analysed to determine the numerical outcomes A(L) for each level using any numerical method’s algorithms. The final numerical value AI for all levels can be then determined by using the following weighted equation:

(12) $$\begin{array}{cc} \upsilon_{n}= & A\left(n+1\right)+\upsilon_{n-1}\times \left(255-A\left(n+1\right)\right)/255\\ \; & \upsilon_{o}=A\left(1\right),\;AI=\upsilon_{M-1} \end{array}$$

In this work the moments of the average value of differences (AVD) is used, where the first $A_{AVD_{1}}\left(L\right)$ and the second $A_{AVD_{2}}\left(L\right)$ moments can be calculated from COM_*L*_ matrix at each level *L* using the following equations [35]:

(13) $$\begin{array}{ccc} A_{AVD_{1}}\left(L\right) & = & \sum_{ij}\frac{COM_{L}\left(i,j\right)}{\sum_{rs}COM_{L}\left(r,s\right)}\left|i-j\right|\\ A_{AVD_{2}}\left(L\right) & = & \sqrt{\sum_{ij}\frac{COM_{L}\left(i,j\right)}{\sum_{rs}COM_{L}\left(r,s\right)}{\left(i-j\right)}^{2}} \end{array}$$

**Figure 3:**
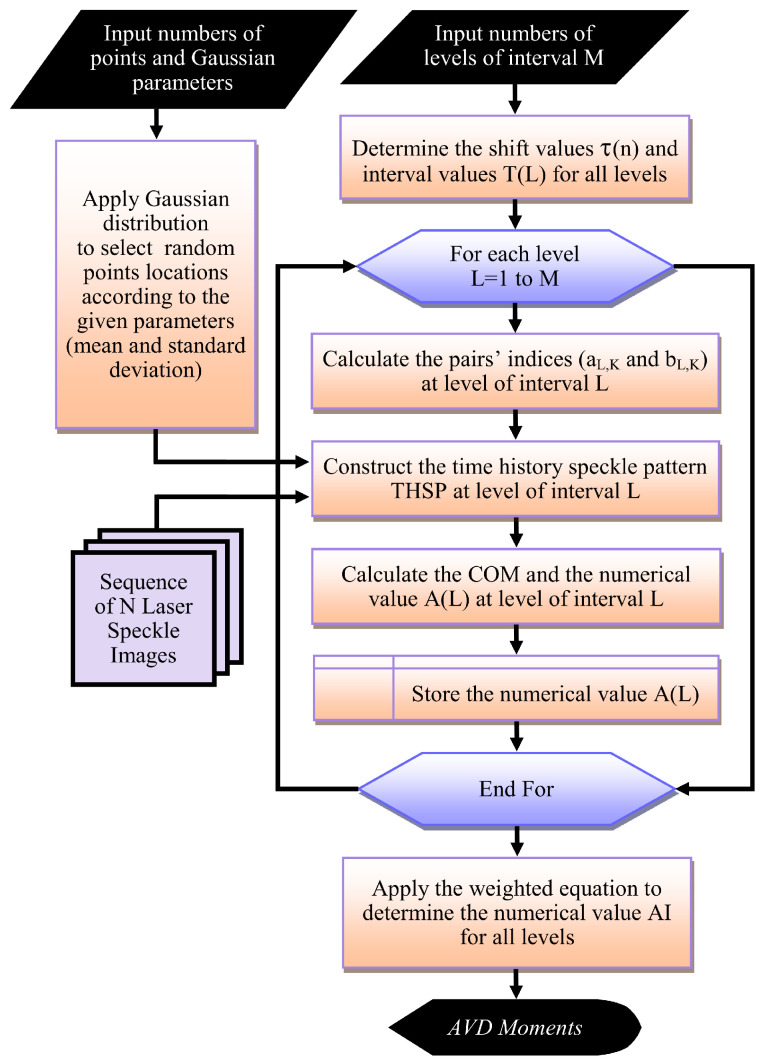
The general steps used by the proposed adaptive levels of interval methodology to improve the performance of the numerical methods.

The general steps used by the adaptive levels of interval methodology to improve the numerical methods’ performance can be summarized as shown in the flowchart of Figure 3. These general steps include:

1. Identify the number of points used to construct the THSP matrix, the Gaussian distribution parameters (mean and standard deviation), and the level of interval number *M* (should not exceed $\left\lfloor \log_{2}\left(1+N/2\right)\right\rfloor \in Z^{+}$).
2. Similar to the second step of the graphical methods. This step determines the shift values τ(n) and the interval value T(L) for all levels.
3. Calculate the A(L) at each level of interval *L* by performing the following operations:
  - Using the pre-calculated shift and interval values with Equations (3) and (4) to calculate the pairs’ indices ($a_{L,K}$ and $b_{L,K}$) at the level of interval *L*.
  - Constructing the THSP_*L*_ at each level *L* from a set of image pairs of the indices ($a_{L,K}$ and $b_{L,K}$) using the generated random locations for the selected Q points.
  - Calculating the co-occurrence matrix COM_*L*_ at *L* using Equation (11) and determining with storing its numerical value A(L) using selected algorithm equation (like AVD numerical algorithm’s Equation (13)).
4. Apply the weighted Equation (12) to determine the final numerical value AI for all levels to quantify the activity or level of changes in specific speckle regions.

Since the adaptive levels of interval methodology solely depend on the available number of images used to record speckle pattern phenomena to adapt the levels of interval. This makes changing the speckle pattern scales of activity and their physical characteristics (like size, intensity, etc. results from the laser source and the scattered object parameters) do not have any impact on the stability of the interval adjustment.

## 3. Evaluation Procedure and Results

This section presents the evaluation procedure and results for the graphical and numerical tests used to evaluate the impact of the proposed methodology on the processing performance of graphical and numerical methods, respectively.

### 3.1. Graphical Evaluation

Evaluating the impact of the proposed ALI methodology on the graphical methods’ performance is implemented by generating and processing synthetic correlated patterns of laser speckles with various parameters, including different activity scales and granular speckle sizes.

#### 3.1.1. Generation of Correlated Speckle Patterns

**Figure 4:**
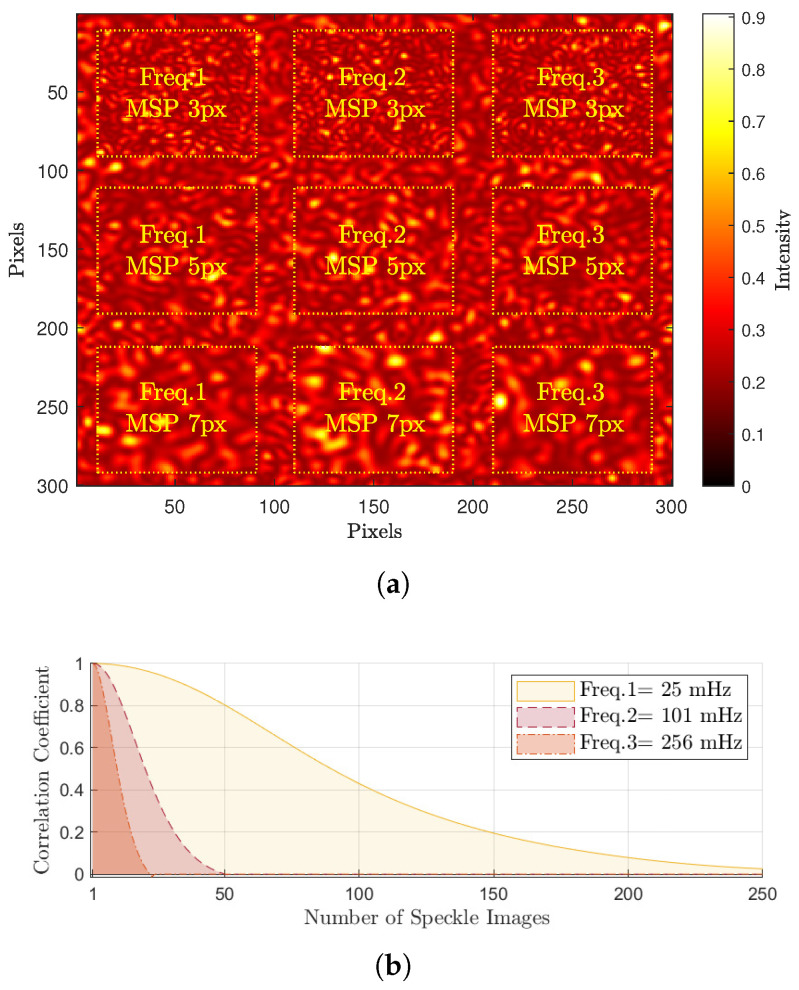
Synthetic laser speckle image with speckle correlation coefficients at different frequencies. (**a**) Generated speckle image at a given time of evolution. (**b**) Speckle correlation values at different frequencies.

Accurate graphical evaluation requires the generated speckle images to have regions of dynamic and static speckle synthetic patterns with various activity scales and granular speckle sizes. This was implemented in this test by generating nine sequences of speckle patterns having different parameters and combining these sequences with a static speckle patterns sequence at predefined locations and widths, as shown in Figure 4a.

This figure presents a single generated speckle image having a static background pattern with nine square regions of dynamic speckles patterns of different granular sizes defined by minimum speckle size MSP in pixel units px (3, 5, and 7 px) and different evolution frequencies of activity scales Freq.1→3 (corresponding to 25, 101, and 256 mHz respectvely).

In this work the nine sequences were generated using an efficient modelling tool for simulating speckle dynamic phenomena called the copula algorithm [43]. Referring to reference [33], this algorithm synthesizes 600 band-limited correlated speckle patterns of a predefined correlation’s evolution for each sequence by creating 600 (dxd) matrices having a (*D* diameter) circular region of complex numbers with unity amplitude and randomly distributed phases between (0,2π) [33,43,44].

The phase arrays for these regions $\phi_{k},k=1,2,\cdots ,N$ were determined by applying the percentile transformation using the following equations [33,43,45]:

(14) $$\phi_{k}=2\pi F_{Z}\left[\sqrt{-2\ln X_{1}}\cos\left(2\pi X_{2}+\frac{\pi }{2}\frac{k-1}{N-1}\right)\right]$$

where N is the number of generated patterns, $F_{Z}$ is function of normal cumulative distribution, and $X_{1,2}$ are independent uniformly distributed random variable [33].

The complex matrices are then transformed by Fourier transform and multiplied by their complex conjugates, where the d/D ratio is used to adjust the minimum size for the speckle patterns MSP [33,46]. According to the Nyquist criterion, this ratio should be greater or at least equal to two pixels for good speckle pattern representation [33,43,46].

Figure 4b shows the calculated correlation coefficients for dynamic speckle regions of different evolution frequencies, where these coefficients are continuously calculated at each region by applying a correlation between the initial generated speckle image and the successive generated images that follow it. The rapid decrement in the correlation values with incrementing the values of the evolution frequency validates the efficiency of the generation algorithm to synthesise correlated speckle patterns.

#### 3.1.2. Graphical Evaluation Results

The evaluation results for the impact of the presented methodology on the graphical methods’ performance were obtained by processing the generated speckle patterns with three sets of graphical processing methods. The first set represents the original (I.M.) and modified (ALI I.M.) inertial moment methods. The second set represents the original (Fujii) and modified (ALI Fujii) Fujii methods, while the last set represents two hybrid methods resulting from utilizing the mixing capability for the presented methodology to process the speckle patterns with more than one graphical method. This is through calculating the activity maps for the lower levels with Fujii and the upper levels maps with I.M or vice versa resulting (ALI Fujii-I.M.) and (ALI I.M.-Fujii) respectively.

**Figure 5:**
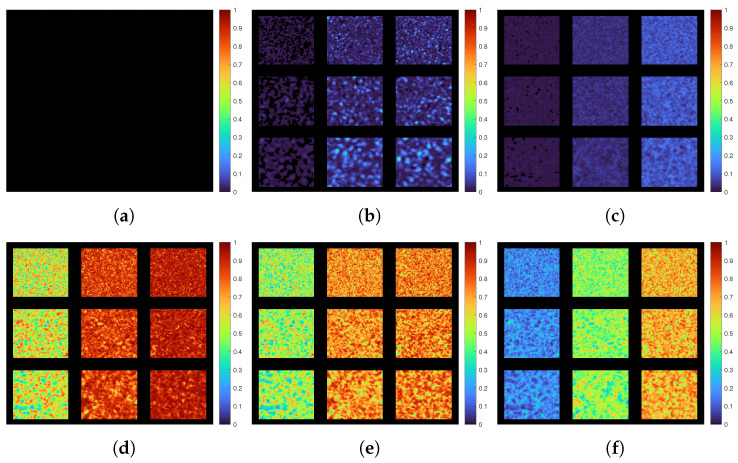
Graphical evaluation results for I.M. and Fujii methods before (**a**,**c**) and after applying ALI methodology on them as individual (**b**,**d**) and combinational (**e**,**f**). (**a**) I.M. Method. (**b**) ALI I.M. Method. (**c**) Fujii Method. (**d**) ALI Fujii Method. (**e**) ALI Fujii-I.M. Method. (**f**) ALI I.M.-Fujii Method.

The resultant activity maps, which represent the graphical outcomes for this evaluation test were presented using pseudo-colour format as shown in Figure 5. This representation format, colours the low activity region with blue colour and high activity region with red colour.

Referring to this figure, the activity maps for the first set of the speckle processing methods show almost no activity values for the original (I.M.) across the entire regions compared to the modified (ALI I.M.) method. In the second set, the results also show an increment in the activity values for the modified (ALI Fujii) method over the original (Fujii) method like the first set’s outcomes but with higher activity values for the speckle processing methods (original and modified) of the second set. Regarding the last method set of using the adaptive methodology to combine both (I.M.) and (Fujii) methods, the graphical outcomes show high activity values for all dynamic speckle regions for both combinations (ALI I.M.-Fujii, FUjii-I.M.). These results also show different activity distributions corresponding to the evolution frequencies of these regions, with wider distribution for (ALI I.M.-Fujii) compared to (ALI Fujii-I.M.) method.

### 3.2. Numerical Evaluation

Evaluating the impact of the presented methodology on the activity extraction capability for the numerical processing methods (presented in the Section 2.2) is implemented in this test. This is by generating a set of one hundred complex signals that emulate the scattering intensities of speckle interference phenomena at different scales of activity. This is by creating at each activity scale a composite signal consisting of two sinusoidal waveforms of different amplitudes and frequencies using the following equation:

(15) $$CS=\left[\underset{1st\;Sinewave}{\underset{︸}{\left(0.9\times \sin(2\pi \;0.2\rho t)\right)}}+\underset{2nd\;Sinewave}{\underset{︸}{\left(0.1\times \sin(2\pi \;\rho t)\right)}}+1\right]/2$$

where ρ is the activity factor, its values ranging from [0→1] with precision incremental equal to 0.01.

This makes the frequency values for the 1stsinusoidal of 0.9 amplitude and 2ndsinusoidal of 0.1 amplitude ranging between [0→0.2Hz] and [0→1Hz] respectively.

The evaluation test then applies these signals to extract the numerical values for their activity scales using the first and the second AVD moments with and without applying the proposed methodology of constructing the time history of the speckle pattern THSP at different levels of interval. In this test, the modified term is used to describe the numerical methods that result from applying the proposed methodology.

**Figure 6:**
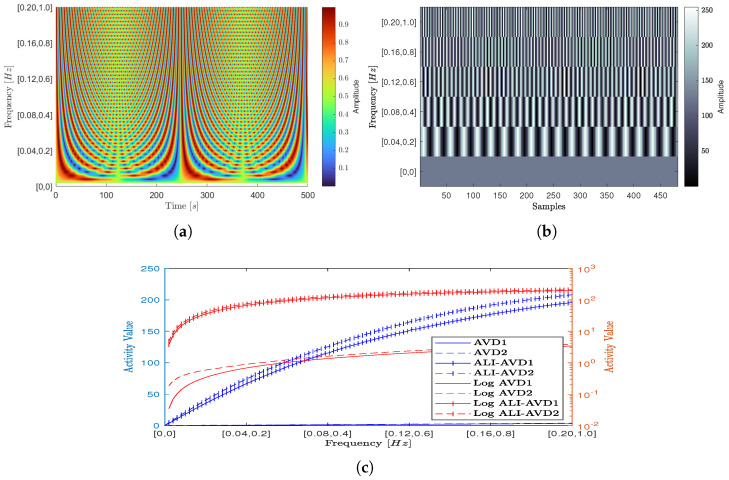
Different scattering intensity signals with selected samples of the THSP and the numerical evaluation results before and after applying ALI methodology on AVD moments. (**a**) Composite signals with different frequencies. (**b**) THSP at different frequencies combinations. (**c**) Activity values in linear and logarithmic scales.

Figure 6, shows scattering intensity signals (Figure 6a) used in this evaluation plotted along the time axis at one hundred activity factors. These factors are represented at the frequency axis with the corresponding frequencies of their values [1st−termFreq,2nd−termFreq.]. The figure also shows selected samples of the calculated THSP (Figure 6b) at six frequency combinations. The numerical evaluation results of activity values are also presented in this figure for the traditional (AVD1, AVD2) and modified (ALI-AVD1, ALI-AVD2) numerical processing methods (Figure 6c) in both scales linear and logarithmic.

Referring to Figure 6, the numerical evaluation results show a comparable performance between the AVD1 and AVD2 in both scales (linear and logarithmic) with low responsivity values across the entire range of frequencies. The results also show an increase in the responsivity and the sensitivities for both moments’ values when applying the proposed methodology of constructing the THSP at different levels of interval, with slightly higher responsivity for ALI-AVD2 over ALI-AVD1.

## 4. Experimental Validation

The effectiveness of the proposed methodology on the graphical and numerical speckle processing’s performance presented in the Section 3 can be validated experimentally by acquiring speckle patterns at different activity scales and processing them graphically and numerically using the presented methodology.

This section illustrates the design and implementation of an exceptional laser imaging system used to generate the required speckle patterns for experimental validation. The section then illustrates the procedure of validation and presents the experimental validation results with a discussion about them.

### 4.1. Laser Speckle Imaging System

One of the major aspects that should be considered during the experimental validation is the need for tests that give a universal understanding about the processing performance of the presented methods without and with applying the adaptive methodology. This is because the most established graphical and numerical methods in the literature selected in this work are used in diverse fields of applications, which makes their evaluation mainly related to the types of these applications.

These applications have unique experimental setups and the interpretation of their graphical or numerical outcomes mainly depends on targeting parameters of specific interference phenomena in the scattering medium. This makes the application-based evaluation lose generality and raises the demand towards application-independent evaluation.

Implemented application-independent evaluation for the processing method can be done by generating experimentally dynamic speckle patterns similar to what all applications provided at the acquisition stage but with adjusted parameters linked to their scales of activity directly, not to the interference of scattered wavelets in the medium of a specific application.

**Figure 7:**
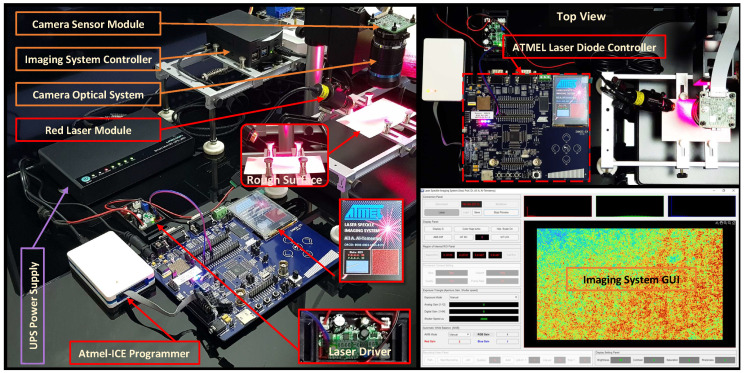
Imaging system speckle generation, speckle acquisition, power supply, and GUI controlling units and their hardware components.

The laser imaging system used in the experimental validation is developed in an exceptional manner to generate sequences of dynamic laser speckle patterns with adjusted scales of activity suitable for providing application-independent evaluation with high accuracy. The architecture of the implemented imaging system and its hardware components are shown in Figure 7. This architecture consists of four units these are:

- Dynamic Speckle Generation Unit: This unit performs the dynamic laser speckle pattern creation at a controllable scale of dynamic activity with high accuracy. It is an exceptional unit in the speckle imaging system and is vital to the experimental validation. It comprises three main components:
  ⋄ Laser Illumination Module: This module consists of a red-laser module (MTOLASER) and a 12 V driver (Micost-optotech, Shenzhen). The laser-module contains a 100 mW–660 nm semiconductor laser attached to a beam expander optical kit. The expanded laser beam for this module was directed with a specific angle towards a diffuse rough surface to form fully developed speckle patterns and detour specular reflection.
    The generation of these patterns can be controlled through the driver. This driver is used to derive the laser module and adjust its input current according to the modulated frequency of the Pulse Width Modulation (PWM) input signal, which varies between 1 KHz and 15 KHz.
  ⋄ Atmel Laser Diode Controller: It is an embedded processing platform (Atmel, CA, USA) based on combining ARM^®^ Cortext^®^-M3 processor, storage units, interface peripherals and input/output components including input switches and graphical display on a single board.
    This platform is responsible for controlling the speckle generation process by executing a special firmware able to communicate with its interface output port to provide the laser driver with the required PWM signal.
    This firmware has been developed with its graphical interface (shown in the platform display unit) to generate a triangular waveform of frequency that varies between 0.02 Hz and 0.35 Hz. It uses this waveform to modulate the duty cycle of a PWM 5 KHz signal with a resolution equal to one thousand divisions and duty cycle value varies between 450 and 1000 to keep the driving input current for the laser module fluctuating above the value of the lasing threshold.
  ⋄ Atmel ICE Programmer: The ICE is a programming tool that uses the Atmel software framework (ASF Version 3) to write the firmware for the Atmel laser controller. In addition, this tool is used to develop the graphical user interface for this firmware to display the parameters required to control the scales of activity for the generated speckle patterns and also the 2D-plot of duty cycle modulated waveform in real-time (bottom centre of Figure 7).
- Dynamic Speckle Acquisition Unit: The main task of this unit is to detect and register the interference speckle patterns of backscattering laser wavelets generated by the speckle generation unit. The main components of the acquisition unit are the camera sensor with its optics and the imaging controller.
  ⋄ The Camera Sensor Module: The module used in this unit adopts the technology of high-speed image registration at low noise and high sensitivity. It is based on a diagonal 7.857 mm imaging sensor from Sony-IMX477 with a spatial resolution reaching 12.3 MP. An optical system is attached to this module to detect the subjective speckle patterns, which consists of a 16 mm lens and F 1.4–16 aperture. A vertical stage with adjusting capability is used with the optical system to add another degree of freedom during the focusing process.
  ⋄ The Imaging System Controller: It is an embedded controlling platform that uses a 1.5 GHz Quad-core ARM processor with dedicated interface ports to enable the Graphical User Interfaces (GUI) controlling unit to communicate with the camera sensor module. It uses MIPI Camera Serial Interface and Ethernet interface ports to control the sensor module with the incoming command from the GUI controlling unit and transfer the registered images of speckle patterns to this unit.
- Power Supply Unit: This unit provides stable operation for the dynamic speckle generation and acquisition units. This is through supplying the components of these two units with high-accuracy regulated voltages and currents.
- GUI controlling Unit: This unit executes a special GUI software (MATLAB R2023b) to control the process of dynamic laser speckle generation and acquisition. This software was developed for the imaging system to enable users to select the required parameters related to speckle generation and acquisition during the experimental validation.

### 4.2. Validation Procedure

The procedure of validating the effectiveness of the proposed methodology on the processing performance of the graphical and numerical methods comprises two experimental validation tests, dynamic speckle patterns and processing speed tests.

#### 4.2.1. Dynamic Speckle Patterns Test

This validation test utilizes the unique feature of the designed imaging system. It uses the dynamic speckle generation unit to create multiple sequences of dynamic speckle patterns at a wide range of activity scales through adjusting the evolution’s frequency parameter for this unit with values between [0.02 Hz → 0.35 Hz]. During the generation process, the dynamic speckle acquisition unit was used to register the generated sequences and transfer them to the GUI controlling unit, which stores them according to their activity scale values. The sensor and optical-setup parameters used with the imaging system (like frame rate, image resolution, illumination angle, aperture, and optical distance from the illuminated surface) were adjusted to obtain 1600 fully-developed speckle patterns for each sequence with high temporal and spatial resolutions satisfy the Nyquist criterion.

**Figure 8:**
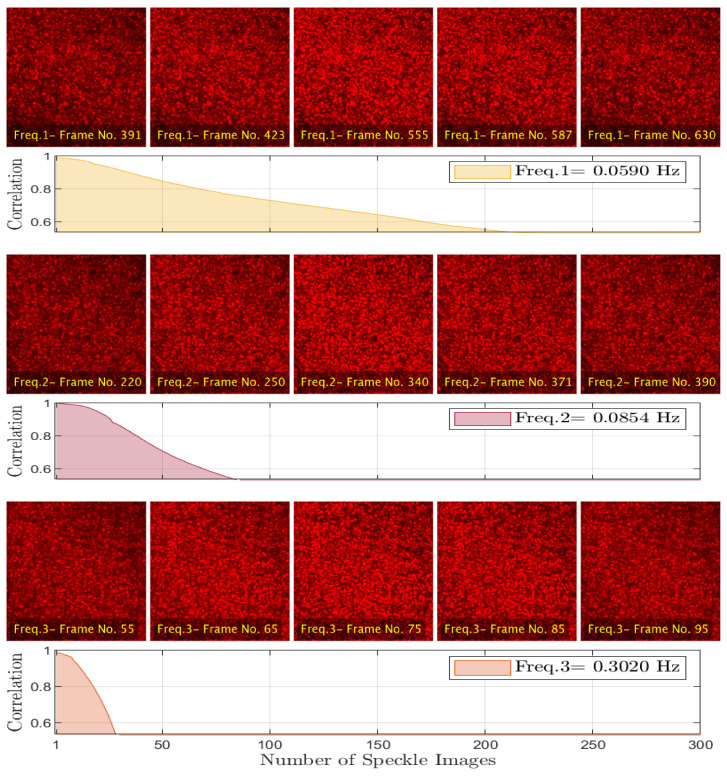
Generated speckle sequences and their correlation coefficients.

Figure 8, presents three sequences of dynamic speckle patterns that are generated by a laser imaging system with different evolution frequencies. The figure also validates the speckle generation process through demonstrating how the correlation coefficients of these generated sequences are decreased rapidly with increasing the evolution frequencies (scales of activity).

The generated sequences of speckle images which reach to forty thousand images were then processed in this test by three sets of graphical processing methods (presented in Section 3.1.2) and one set of numerical processing methods (traditional and modified presented in Section 2.2), which leads to processing more than three hundred thousand speckle images. The activity values for each type of these methods (graphical and numerical) are then plotted across the generated sequences’ frequencies, where the averaging process on the activity map is used with the graphical methods to represent their results on a 2D plot like the numerical methods’ outcomes.

#### 4.2.2. Processing Speed Test

The impact of modifying the graphical (Fujii and I.M.) and numerical (THSP-based) methods on the processing speed performance (which represents a vital parameter in laser speckle imaging) is evaluated in this test. This is by generating speckle sequences of different image numbers and processing them with measuring the processing time required at each number of images. To increase the processing-speed evaluation accuracy, the whole procedure for this test is repeated ten times and the average processing time and standard deviation values at each number of images are then determined. The testing parameters including generated speckle images, programming language, and computing platform are: 1066 × 1087 pixels spatial resolution, MATLAB (R2023b) software, 64-bit Microsoft Windows 10 operating system, Lenovo-Legion 5 (15IMH05) Laptop, 16 GB RAM, and Core^™^ i7-10750H Intel^®^ 2.6 GHz processor.

### 4.3. Validation Results and Discussion

#### 4.3.1. Dynamic Laser Speckle Patterns

The validation test of processing generated speckle sequences of different evolution frequencies produces two validation results types, one related to the graphical methods’ sets and the other to the numerical processing method’s set.

**Figure 9:**
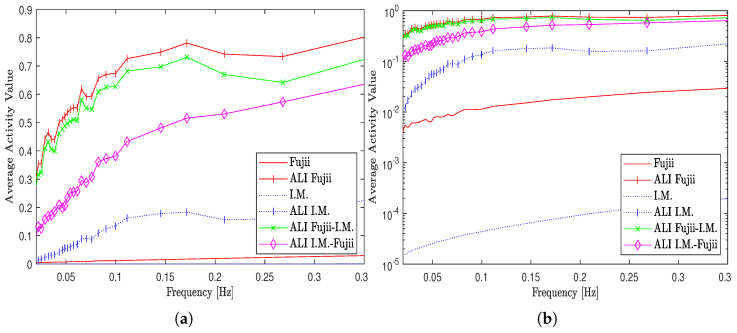
Average values of activity of the dynamic speckle test for three sets of graphical speckle processing methods including original, modified, and hybrid. (**a**) Average activity values in linear scale. (**b**) Average activity values in logarithmic scale.

Regarding the graphical processing methods, the validation results (average activity values) for the first (original I.M. and modified ALI I.M. inertial moment methods), second (original Fujii and modified ALI Fujii methods), and the last set (ALI Fujii-I.M. and ALI I.M.-Fujii hybrid methods) are presented in linear and logarithmic scales as shown in Figure 9.

**Figure 10:**
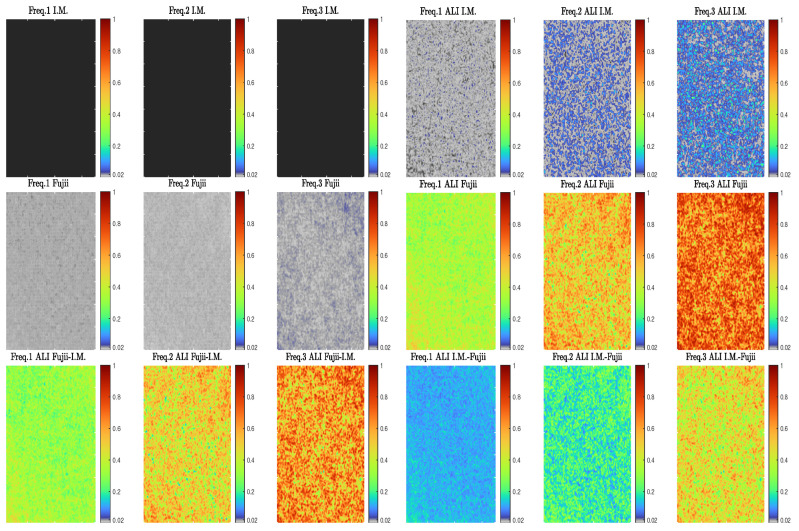
Selected activity maps for different graphical method types at three selected values of evolution frequency (Freq.1 = 0.0590 Hz, Freq.2 = 0.0854 Hz, and Freq.3 = 0.3020 Hz).

Since the presented validation values in Figure 9 were calculated from applying an averaging process on the resultant activity maps of graphical methods’ sets. Figure 10, shows some of these maps for each processing method type at three selected values of evolution frequency (Freq.1 = 0.0590 Hz, Freq.2 = 0.0854 Hz, and Freq.3 = 0.3020 Hz), where two map types were used to represent this figure efficiently, Gray-scale map for small values [0→0.01] representation and Pseudo-colour map for the rest of the activity values.

Referring to Figure 9a,b, the average activity values show low responsivities for the original I.M. and Fujii, where the former is lower in rank than the latter. Regarding the modified versions (ALI I.M., ALI Fujii) of these methods when the adaptive methodology was used, the results show high responsivity values for them in comparison with those of the original versions. The figure also shows high responsivity values for the hybrid methods (ALI Fujii-I.M., ALI I.M.-Fujii) with different sensitivities across the entire range of frequencies.

**Figure 11:**
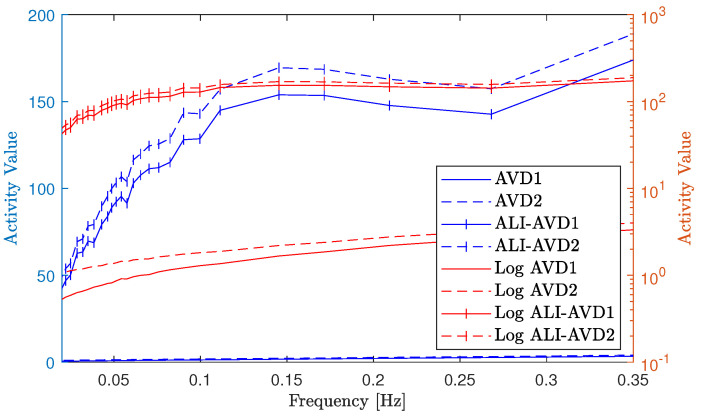
Dynamic speckle activity values for traditional and modified numerical methods.

Regarding the numerical processing methods, the validation activity values for the traditional (AVD1, AVD2) and modified (ALI-AVD1, ALI-AVD2) numerical processing methods are presented in Figure 11 in linear and logarithmic scales.

Referring to this figure, the validation results show comparable values of activity between the traditional (AVD1, AVD2) numerical methods, where the values of their responsivity are low across the entire frequency range (scales of activity). The results also show an increase in both responsivity and sensitivities (especially before 0.15Hz) for the modified (ALI-AVD1, ALI-AVD2) methods result from applying the proposed methodology of adaptive levels of interval, with slightly higher responsivity for ALI-AVD2 over ALI-AVD1.

**Figure 12:**
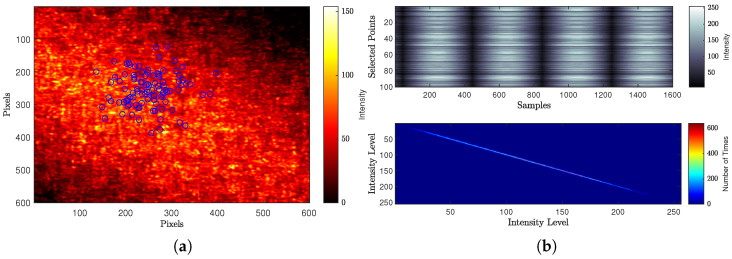
Activity value calculation using traditional numerical method. (**a**) Selected points of the THSP on one of the speckle pattern images. (**b**) THSP and COM for the traditional numerical method.

**Figure 13:**
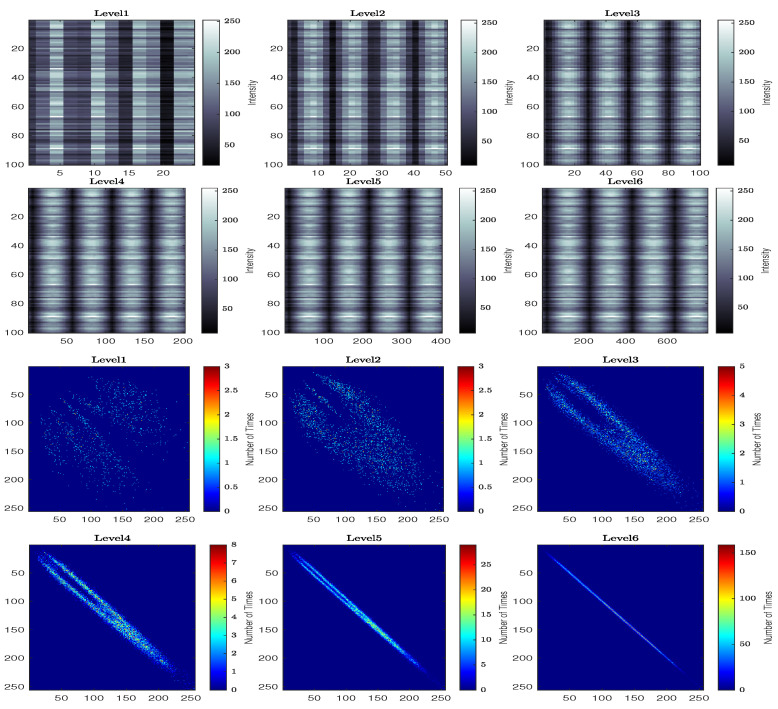
Activity value calculation using modified numerical method.

Figure 12 and Figure 13 demonstrate how the generated speckle patterns are processed by the traditional and modified versions of numerical speckle processing methods to determine the activity values. Figure 12a, shows the Gaussian-distributed locations for randomly selected points of the THSP on one of the speckle pattern images, while Figure 12b shows the constructed two-dimensional THSP matrix from these points and its intensity dispersion analysis using co-occurrence Matrix (COM).

Regarding the modified numerical method that is used the proposed methodology to construct the THSP matrices at interval levels of different sensitivity scales with the ongoing process activity. Figure 13, shows the constructed THSP matrices (in pseudo-colour representation) for six levels of interval (Level1→6) with their co-occurrence matrices, where the colour-bars for THSP and COM represent the intensity values and the number of times (co-occurrence) respectively.

#### 4.3.2. Processing Speed

**Figure 14:**
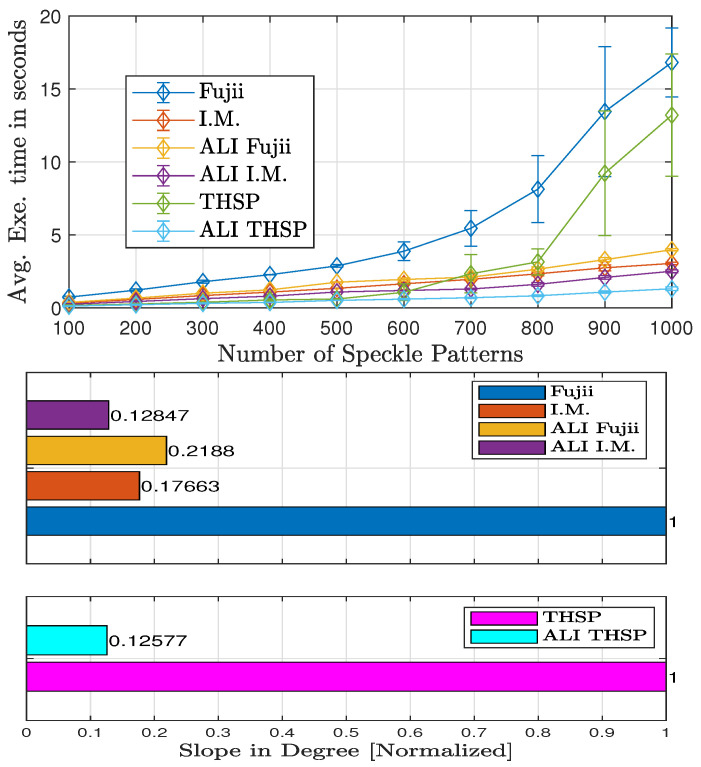
Average execution times with standard deviation (**Top**) and slopes for the Graphical and Numerical (**Bottom**) processing methods without and with applying ALI methodology.

The evaluation results for the processing speed performance that reflect the impact of modifying the graphical (Fujii and I.M.) and numerical (THSP-based) methods using ALI methodology are presented in Figure 14. This figures shows the average execution-time (curved lines) and the standard deviation values (error bars) among different numbers of patterns, in addition, it also provides a quantitive comparison through presenting the normalized slope values of these curves for graphical and numerical methods on separate graphs. This is through applying the least square method on the execution-time curves, which represents an efficient tool for slope value calculation [47].

Referring to Figure 14, the execution-time slope results for the graphical methods show a large slope value for Fujii compared to the other slopes, where this value decreases approximately to 78% when ALI methodology applied (see slope of ALI Fujii), which reflect increasing processing speed. Similar behaviour with I.M slope, which also decreases approximately to 27% with applying ALI methodology (see slope of ALI I.M.) but this decrement is smaller than that of Fujii. Regarding the numerical methods, the execution-time slope values also show a decrement with applying ALI methodology, in which the THSP slope value decreases approximately to 87% (see ALI THSP slope).

## 5. Conclusions

Laser speckle imaging is the field of imaging the interference laser speckle patterns and extracting their spatial and temporal information. The advancement in sensing technology for registering the successive intensity of these patterns makes the intensity-based graphical and numerical processing methods preferable for extracting the speckle-embedded information.

In this paper, the Adaptive Level of Interval ALI Methodology has been developed for use in the laser imaging field to improve the extraction and discrimination capabilities for graphical and numerical methods when processing dynamic speckle patterns. This methodology enables these methods to adapt the levels of speckle images interval during processing to extract the activity information through determining and combining at these levels the activity maps and THSP matrices having different sensitivity scales with the ongoing process activity.

The theoretical background and mathematical derivations were presented for using the new methodology with standard graphical methods and numerical algorithms like I.M., Fujii, and AVD moments. The methodology’s impact on the imaging methods’ performance was evaluated using graphical and numerical evaluation tests, in addition, an imaging system was developed to undertake a series of application-independent experimental validation tests. The imaging system architecture with its hardware components and the procedure of experimental validation including dynamic speckle patterns and processing speed tests were presented.

The evaluation results of using correlated speckle patterns and composite signals show better extraction and discrimination performance for the graphical and numerical methods with using ALI methodology and prove its ability to provide different scales of responsivity and sensitivity through combining multiple graphical methods. Similar behaviour was shown in the experimental results, which proves the efficacy of this methodology on the extraction and discrimination capabilities of imaging methods and validates its ability to provide high performance. Regarding the processing speed test, the decrement in the execution-time slopes for both graphical and numerical methods with using ALI methodology reflects the processing speed improvements and proves the efficacy of organising the adaptable pairs’ intervals with non-overlapping structure to evade redundant usage of the speckle images.

Presenting the general formulas and implementation steps with its flowcharts to improve the graphical and numerical speckle processing methods using ALI methodology makes its use not restricted to the presented methods in this work, but also with any type of consecutive graphical and THSP-based numerical processing methods. This in turn opens up the possibility towards using this methodology for improving various processing methods in the laser speckle imaging field.

## Data Availability

The raw data supporting the conclusions of this article will be made available by the author on request.
